# Effects of Propolis Impregnation on Polylactic Acid (PLA) Scaffolds Loaded with Wollastonite Particles against *Staphylococcus aureus*, *Staphylococcus epidermidis*, and Their Coculture for Potential Medical Devices

**DOI:** 10.3390/polym15122629

**Published:** 2023-06-09

**Authors:** Ana Isabel Moreno, Yeison Orozco, Sebastián Ocampo, Sarita Malagón, Alex Ossa, Alejandro Peláez-Vargas, Carlos Paucar, Alex Lopera, Claudia Garcia

**Affiliations:** 1 Grupo de Cerámicos y Vítreos, Universidad Nacional de Colombia sede Medellín, Medellín 050034, Colombia; 2 Faculty of Dentistry, Universidad Cooperativa de Colombia sede Medellín, Medellín 055422, Colombia; 3 School of Applied Sciences and Engineering, Universidad Eafit, Medellín 050022, Colombia; 4 Grupo de Nanoestructuras y Física Aplicada (NANOUPAR), Universidad Nacional de Colombia, La Paz 202017, Colombia

**Keywords:** scaffolds, propolis, antibacterial activity, TPMS, additive manufacturing

## Abstract

Several diseases and injuries cause irreversible damage to bone tissues, which may require partial or total regeneration or replacement. Tissue engineering suggests developing substitutes that may contribute to the repair or regeneration process by using three-dimensional lattices (scaffolds) to create functional bone tissues. Herein, scaffolds comprising polylactic acid and wollastonite particles enriched with propolis extracts from the Arauca region of Colombia were developed as gyroid triply periodic minimal surfaces using fused deposition modeling. The propolis extracts exhibited antibacterial activity against *Staphylococcus aureus* (ATCC 25175) and *Staphylococcus epidermidis* (ATCC 12228), which cause osteomyelitis. The scaffolds were characterized using scanning electron microscopy, Fourier-transform infrared spectroscopy, differential scanning calorimetry, contact angle, swelling, and degradation. Their mechanical properties were assessed using static and dynamic tests. Cell viability/proliferation assay was conducted using hDP-MSC cultures, while their bactericidal properties against monospecies cultures (*S. aureus* and *S. epidermidis*) and cocultures were evaluated. The wollastonite particles did not affect the physical, mechanical, or thermal properties of the scaffolds. The contact angle results showed that there were no substantial differences in the hydrophobicity between scaffolds with and without particles. Scaffolds containing wollastonite particles suffered less degradation than those produced using PLA alone. A representative result of the cyclic tests at F_max_ = 450 N showed that the maximum strain reached after 8000 cycles is well below the yield strain (i.e., <7.5%), thereby indicating that even under these stringent conditions, these scaffolds will be able to work properly. The scaffolds impregnated with propolis showed a lower % of cell viability using hDP-MSCs on the 3rd day, but these values increased on the 7th day. These scaffolds exhibited antibacterial activity against the monospecies cultures of *S. aureus* and *S. epidermidis* and their cocultures. The samples without propolis loads did not show inhibition halos, whereas those loaded with EEP exhibited halos of 17.42 ± 0.2 mm against *S. aureus* and 12.9 ± 0.5 mm against *S. epidermidis*. These results made the scaffolds possible bone substitutes that exert control over species with a proliferative capacity for the biofilm-formation processes required for typical severe infectious processes.

## 1. Introduction

Several tissues of the human body require partial or total regeneration or replacement owing to different diseases and injuries that cause irreversible damage. Bones are the second-most transplanted tissue in the world, with more than four million surgeries per year performed using bone grafts or substitutes [1]. Conventional regeneration techniques involve the transplantation of a living bone tissue either from one part of the body to another in the same patient (autograft), from one individual (living or dead) to another (allotransplant or allograft), or even from animals (xenograft); the latter presents low use expectations owing to its scarce utility for medical needs and other complications [2]. Nowadays, tissue transplants from the same patient are the most widely used and recommended technique because they offer all the necessary properties for a bone-graft material. However, its success probability depends on multiple factors that, until now, have been difficult to overcome, including insufficient tissues in the patient for the affected area, bones with an insufficient functional state in the donor area, and the requirement for other surgical procedures to promote graft regeneration, thereby increasing the treatment expenses and the probability of causing new injuries and morbidity [3]. To mitigate the largest number of problems, bioengineering and materials science have been used to advance toward tissue engineering or regenerative medicine, whose aim is to develop biological substitutes to restore and/or improve natural tissue functions by cell and tissue stimulation and manipulation. In this sense, considerable progress has been reported in this field in the last 20 years [2]. Tissue engineering combines three-dimensional (3D) lattices (scaffolds) of cells with biologically active molecules and growth factors that promote cell evolution to create functional bone tissues [4]. To guarantee their functionality, the architecture, composition, and properties of the scaffolds must be reasonably similar to the original extracellular bone lattices because cells will develop within this environment until they form their own lattice and generate a new replacement tissue [5]. 

Additive manufacturing is one of the most visible and usable tools for manufacturing implantable devices because its layer-by-layer design facilitates controlling baseline characteristics such as pore size, interconnectivity, and complex geometries, which may be impossible using traditional manufacturing [6]. Three-dimensional printing has received considerable attention because of its high degree of control over the macro-architecture and microarchitecture of porous tissues [7]. However, although considerable advances have been reported, bone implants are still affected by bacterial infection, specifically osteomyelitis, which causes 2–5% of total transplant patients to undergo surgery again to replace implants and treat infections [8]. 

There is a wide range of biomaterials used to manufacture implantable devices, including wollastonite (CaSiO_3_), a calcium silicate whose characteristics have turned it into a leading material used for bone-regeneration devices [9]. Wollastonite has also demonstrated bioactivity, biocompatibility, and antibacterial properties [10,11,12]. Moreover, polylactic acid (PLA) is a biodegradable, thermoplastic aliphatic polyester characterized by its biosafety, good biocompatibility, and low toxicity, which is why it was approved by the U.S. Food and Drug Administration as a human biomedical material [13]. Until now, PLA has been used to develop a large number of items for orthopedics and dentistry, such as screws, fixation devices, and others [14,15]. Despite its good properties, adding particles to PLA can improve its mechanical and osseointegration properties [16]. 

Due to its characteristics, additive manufacturing is an appropriate technique for manufacturing scaffolds. Notably, 3D printing includes several techniques such as fused deposition modeling (FDM), fused filament fabrication [17,18,19], stereolithography, and selective laser sintering. Developed in 1996, FDM was one of the first 3D printing techniques. It involves the extrusion of a fused material deposited layer by layer by following software-programmed instructions [20]. Nowadays, FDM is one of the most popular techniques because it is easy to handle and quite affordable. Generally, FDM is used with thermoplastic materials, which allows each layer to be redirected upon being deposited, and the control of space between paths, thus creating scaffolds with uniform internal structures and managing pore interconnection, as well as their size and morphology [21]. These characteristics are crucial aspects for designing and manufacturing scaffolds as a part of tissue engineering applied to tissue repair processes. 

Materials and techniques are not the only critical factors in designing bone-insertion devices. The geometries used are also critical to facilitate cell proliferation and healing processes. Within this context, triply periodic minimal surfaces (TPMSs), minimum-area surfaces limited by a closed curve, emerge. A unit cell is repeated a definite number of times to form TPMS-based scaffolds. The unit cells are combined, and a thickness is then added to the surface to generate a symmetrical 3D figure [22]. The complex geometries of TPMSs suggest their potential use in scaffolds because they can closely mimic natural cortical bone tissues, facilitate a good nutrient transfer, and foster stress shielding [23]. A large number of TPMSs are available, but in bone-regeneration studies, three stand out: gyroid, Schwarz primitive, and Schwarz diamond [24]. Gyroid TPMSs exhibit favorable characteristics for cell growth [22,23,24,25].

Propolis is a resinous substance produced by bees using various waxes, gums, pollen, and sap found in the plants around their hives. The substance is modified by bee secretions to form a viscous compound that bees use to insulate their hives from the cold and protect them from certain pests [26]. The flora surrounding the hive directly influences the propolis properties, with the properties varying with the geographical area in which the hive is located [27,28]. The study of various propolis from several regions has allowed the creation of a general composition profile, which is as follows: gum and amber resin (50–70%), oil and wax (30–50%), and pollen (5–10%). We can also find vitamins B, C, and E, minerals, sugars, flavonoids, phenol amino acids, as well as aromatic compounds [29]. Some propolis has been reported in the literature as potential antioxidant agents, cytotoxic agents, and having antibacterial activity [30,31,32]. Propolis has proven effective against yeasts, different bacterial species, and some parasites. Additionally, it has shown a desirable behavior by forming synergisms that may help control species resistant to conventional antibiotic therapies [33]. Furthermore, until now, no significant toxic effect has been found in animal or human models, thus deeming it a natural option for medical drug development [34]. The antibacterial effects of propolis have been associated with the presence of phenols, flavonoids, and triterpenes, among others [35]. As propolis has shown promising and desirable properties in different fields, including cosmetics, food, and health industries, different technologies have been developed to leverage its full potential. This includes obtaining propolis-loaded alginate beads for gastrointestinal drug treatments [36], its microencapsulation by pectin and soy protein coacervates to leverage its antioxidant and antibacterial properties in food systems [37], and obtaining polycaprolactone (PCL) loaded with 4% propolis using electrospinning to inhibit *Staphylococcus aureus*, *Staphylococcus epidermidis*, *Bacillus cereus*, *Proteus mirabilis*, and *Escherichia coli* [38]. For these same purposes, impregnation methods have also been used. For example, some studies have obtained cellulose acetate and PCL nanofibers by electrospinning, which were later impregnated with ethanolic extracts of propolis to reveal an inhibitory effect against Gram-negative and Gram-positive bacteria, as well as a promising antioxidant effect in providing suitable surfaces for the scar-healing system [39]. Similar studies have also been conducted with PLA filaments but using propolis nanoparticles to impregnate the filaments. These nanoparticles are produced using carboxymethylcellulose, providing antioxidant activity to the filaments and opening the possibility of using them for treating skin wounds [40]. Even with miliQ water, red propolis nanoparticles have been obtained, which have been used to impregnate collagen scaffolds and improve the metabolic activity and cell proliferation of 3T3 fibroblasts [41]. This study aims to obtain PLA and wollastonite scaffolds impregnated with propolis extracts from the Tame, Arauca region (Colombia) and to assess the effects of the presence of propolis in terms of its antibacterial activity against osteomyelitis-causing species, with application possibilities for repairing bone defects. 

## 2. Materials and Methods

### 2.1. Filament Fabrication

Powdered PLA (unitary molecular weight 299.37 g/mol, 150 mesh, Huaian Ruanke Trade Co., Ltd., Huaian, China) was used as the base material to prepare the filaments. Commercial wollastonite particles (NYDA1250 NYCO Minerals, New York, NY, USA) were used as inorganic filler agents. To verify the identity of the crystalline phases in the wollastonite powders, the samples were characterized by X-ray diffraction using a D8 Advance Eco Bruker diffractometer from Bruker (Cu 1.5418 Å, 2θ 10–50°, Karlsruhe, Germany). To obtain filaments of the material loaded with inorganic materials, the PLA and wollastonite powders were mixed (20% *w*/*w* wollastonite in PLA) using a planetary ball mill (Retsch PM 100, Haan, Germany) for 20 min in 5 min cycles with reverse rotation at 250 rpm. This mixture was processed in a wire-drawing extruder (Wellzoom type C, Shanghai, China). The filament was fed through an endless screw at 2000 mm/min, passed through a 1.75 mm nozzle at 160 °C, cooled in a moving water system, and finally recovered through a gear system with an adjustable speed to maintain a constant filament diameter. At the end of this process, a 1.75 ± 0.2 mm filament was obtained.

### 2.2. Collection and Characterization of Propolis Extracts

The propolis came from the Tame region, Arauca (Colombia). All propolis samples were stored at 4 °C before extraction. The extracts of ethanolic propolis (EEPs) were prepared by mixing 10 g of each propolis sample with 100 mL of 70% *v*/*v* ethanol. The mixture was stirred at a controlled speed of 120 rpm for 24 h at 25 °C and filtered. The filtrates were kept in a freezer overnight at −6 °C and then filtered again to remove waxes. The solvent from the solutions was removed using a RE-20000E rotary evaporator at 40 °C and 2.5 bar pressure until one-third of the initial volume of the extracts was obtained.

A colorimetric method was used to quantify the total phenol content in the extracts. Briefly, 20 µL of EEP and 100 µL of Folin–Ciocalteu reagent were added into a 96-well plate. The plate was agitated and set aside for 8 min in the dark. Then, 80 µL Na_2_CO_3_ (0.7 N) was added. The mixture was incubated at 20 °C for 2 h in the dark. The absorbance was measured at 760 nm using a microplate reader (Thermo Fisher Scientific, Waltham, MA, USA). A calibration curve was constructed using concentrations of gallic acid between 50 and 500 µg/mL (r^2^ = 0.998). The results are expressed as mg gallic acid equivalents in g^−1^ EEP (mg GAE g^−1^ EEP). The values are expressed as the mean of three repetitions.

The total flavonoid content of the EEPs was determined using the method reported by Kumazawa et al. [42]. Briefly, 100 µL of EEP and 100 µL of 10% aluminum nitrate (Al(NO_3_)_3_) were added into a 96-well microplate. The plate was agitated and set aside for 1 h at room temperature in the dark. Then, the absorbance was measured at 420 nm using a microplate reader. To prepare the calibration curve (r^2^ = 0.990), concentrations of quercetin (Sigma-Aldrich, St. Louis, MI, USA) between 5 and 350 µg/mL were used. The values are reported as the mean of three repetitions. The results are expressed as mg quercetin equivalents in g^−1^ EEP (mg QE g^−1^ EEP).

### 2.3. Scaffolds Fabrication

The G code used to design the scaffolds was generated using the Ultimaker Cura 4.11.0 software, generating a solid body and creating gyroid fillings without walls and lower and upper layers. Scaffolds with a gyroid-TPMS structure were designed with external geometries shaped like cylinders and parallelepipeds. For degradation and swelling tests, cylindrical geometries with an 8 mm diameter and 3 mm height were used. Mechanical tests were conducted using cylinders of a 13 mm diameter and 5 mm height. Parallelepipeds with a 10 mm × 10 mm square base and 3 mm height were used for other characterization tests.

The scaffolds were 3D printed using an Anycubic Chiron printer with a 0.4 mm outlet-diameter nozzle. A 0.2 mm layer height, 1.0 mm thick walls, 15% filling, and an extrusion temperature of 160 °C were used. The extrusion speed was 25 mm/s, and the cooling fan was maintained at approximately 50% throughout the printing process. No support was used; however, skirts were placed 5 mm from the body to ensure correct filament extrusion.

For the impregnation of the scaffolds with the propolis extracts, 20 μL EEPs were placed on the scaffold surface. These scaffolds were then subjected to a positive pressure chamber (Wiropress, BEGO, San Francisco, CA, USA) for 3 min. Next, we verified if the scaffold had successfully absorbed the extract, and the process was repeated until the total volume absorbed reached 150 μL.

### 2.4. Scaffolds Characterization

#### 2.4.1. Scanning Electron Microscopy

The morphology of the printed scaffolds was observed using scanning electron microscopy (SEM) with a JEOL JSM-7100F device (Tokyo, Japan). The microscope was equipped with a 51-XMX1178 Oxford X-Max energy-dispersive X-ray spectroscope (Abingdon, UK) to observe the scaffolds’ elemental composition and distribution. All samples were metalized with Au–Pd before their observation.

#### 2.4.2. Contact Angle

The hydrophobicity of the PLA samples with and without wollastonite particles was measured by the contact angle formed between a drop of 1 µL of water at room temperature and the surface of the printed pellets. Contact-angle-measuring optical equipment with an OCA 15 EC reference was used to capture the images, and the angles were measured by the ORCA software using 1 µL drops of distilled water. Each sample was measured three times using three drops in different pellet areas, and the corresponding average measurements were reported.

#### 2.4.3. Swelling and Degradation Tests

The swelling rate was measured by immersing dried samples in phosphate-buffered saline (PBS, Sigma, St. Louis, MO, USA) at 37 °C for 4, 8, 12, 24, 28, 32, 36, 48, 52, 56, 60, and 72 h. A paper towel was used to remove excess PBS from the samples. The wet samples were weighed, and the swelling ratio was calculated as per Equation (1) [43,44]:

(1) $$Swelling\;ratio\;\left(\%\right)=\left(\frac{W_{s}-W_{D}}{W_{D}}\right)\times 100,$$

where $W_{s}$ is the weight of the wet sample at a specific time and $W_{D}$ is the weight of the initial dry sample.

The sample degradation ratio was calculated by immersing each scaffold in a 2 mL PBS solution for 7, 14, 21, and 28 days. The samples were weighed on an ADAM AA 160t analytical balance before and after the immersion in PBS. The degradation ratio was calculated using Equation (2):

(2) $$Degradation\;ratio\;\left(\%\right)=\left(\frac{W_{0}-W_{D}}{W_{0}}\right)\times 100,$$

where $W_{0}$ is the initial weight of the scaffolds and $W_{D}$ is the weight of the dry samples after immersion for a given time.

#### 2.4.4. Fourier-Transformed Infrared Spectroscopy

The raw material (PLA powder), PLA filaments with wollastonite particles, and PLA scaffolds with wollastonite particles impregnated with propolis extracts were subjected to a Fourier-transform infrared (FTIR) spectra analysis on a Shimadzu IRTracer-100 with an accessory for measuring the transmittance. The spectra were acquired in a spectral range of 450–2000 cm^−1^.

#### 2.4.5. Differential Scanning Calorimetry

The raw material (PLA powder), filaments with and without wollastonite particles, and scaffolds with and without wollastonite particles were thermally characterized using differential scanning calorimetry (DSC) by employing a DSC-250 from TA Instruments (New Castle, DE, USA). A range of 10–200 °C was used, stabilizing the equipment at 10 °C and using a ramp of 10 °C/min.

#### 2.4.6. Mechanical Response

1. *Monotonic static tests*. Monotonic static compression tests on the scaffolds were conducted using a universal testing machine (Instron 3366, Instron, MA, USA) under a static compression configuration using a force sensor with a maximum range of 3 kN at a speed of 2 mm/min. Force and displacement values were acquired during the test. Five samples for each condition were tested.
2. *Cyclic tests*. Cyclic compressive tests were performed on the scaffolds using a sinusoidal wave of 1 Hz frequency to follow the typical masticatory frequency by employing dynamic testing equipment (TA Electro Force 300) with a load cell of 500 N maximum capacity. Tests were conducted at two different cyclic conditions, depending on the expected maximum load values. First, tests with a maximum load F_max_ = 450 N and a minimum load F_min_ = 45 N, corresponding to an R of 0.1, were performed for up to 8000 cycles to find the maximum deformation in the scaffold. This test condition is considered representative of high masticatory loads, considering that the reported maximum masticatory loads for single occlusions reach up to 1200 N. Moreover, tests with F_max_ = 45 N and F_min_ = 4.5 N were conducted for up to 500 × 10^3^ cycles to get a condition similar to typical masticatory loads during prolonged periods. For each condition, at least five samples were tested.

#### 2.4.7. Antibacterial Activity

1. Bacterial strains. Antibacterial activity was assessed against the following standard strains: *S. aureus* (ATCC 25175) and *S. epidermidis* (ATCC 12228). The strains were previously seeded in Petri dishes containing Mueller–Hinton agar (Merck, Darmstadt, Germany) and incubated at 37 °C for 24 h. To obtain bacterial inocula, the strains were grown to an exponential phase in a brain heart infusion medium (Merck, Germany) at 37 °C for 24 h and adjusted by diluting the new cultures until reaching a turbidity equivalent to 90 NTU (approximately 1.5 × 10^8^ CFU/mL).
2. Determination of the minimum inhibitory concentration (MIC). The MIC was tested for both strains. For this, the pure extract was taken, and serial dilutions were made by taking EEP concentrations ranging from 10% to 0.01% against *S. aureus* and *S. epidermidis*. The obtained inoculum at approximately 1.5 × 10^5^ CFU/mL was then assessed. The inoculum was incubated for 24 h at 37 °C and 5% CO_2_ at different EEP concentrations. The MIC was determined as the final concentration without a visible growth of bacteria.
3. Inhibition zone tests. We used a 90 NTU (approximately 1.5 × 10^8^ CFU/mL) inoculum and the disk diffusion method to assess the antibacterial activity. Twenty microliters of Mueller–Hinton agar medium was poured onto Petri dishes. Each Petri dish was inoculated with a bacterial inoculum. Next, the scaffolds were placed and divided into two groups: the first was impregnated with propolis extracts (150 mg/mL) (CS), and the second was not (US). 0.2% chlorhexidine digluconate and PBS were used as positive and negative control agents. After 24 h of incubation at 37 °C, the inhibition zone was measured in mm using ImageJ; each experiment was performed three times.
4. Biofilm-formation assays for *S. aureus*, *S. epidermidis*, and their coculture. The adhesion assay was performed with each strain and their coculture. 90 NTU (approximately 1.5 × 10^8^ CFU/mL) inocula were used to create two 1:10 dilutions until reaching a 1.5 × 10^6^ CFU/mL concentration for each strain. To prepare the coculture, the two strains were mixed at a concentration of 1.5 × 10^6^ CFU/mL in equal parts. Then, previously sterilized scaffolds were taken, and two groups were formed: the first was impregnated with propolis extracts (CS) (150 mg/mL), and the second was not (US). Both groups were seeded with 1 mL inoculum from each strain and their coculture. The scaffolds were incubated for 24 h and 48 h at 37 °C and 5% CO_2_. After incubation, 3-(4,5-dimethyl-2-thiazolyl)-2,5-diphenyl-2*H*-tetrazolium bromide (MTT, Merck, Germany) was added and incubated for 2 h at 37 °C and 5% CO_2_. The MTT was then removed, and dimethylsulfoxide (Sigma, USA) was added. Finally, a spectrophotometer (Zeiss, Oberkochen, Germany) was used to measure the absorbance at 550 nm.

#### 2.4.8. Cell Viability/Proliferation Assay

Human dental pulp tissues were obtained from third molars extracted for orthodontic reasons. An informed consent was signed to use this biological tissue that would otherwise be discarded. Briefly, chamber pulp was sectioned, and tissue was enzymatically digested using trypsin/collagenase. The primary culture was conducted using D-MEM and contained 10% of fetal bovine serum, 1% of penicillin–streptomycin (100 IU/mL and 2.5 g/mL, Gibco, London, UK), amphotericin B (2.5 g/mL, Gibco), and ascorbic acid (50 g/mL, Sigma). The cultures were incubated at 37 °C in a 5% CO_2_ atmosphere.

Effects of propolis extracts and scaffolds (US and CU) on cell viability/proliferation were evaluated using human pulp-derived mesenchymal stem cells (hDP-MSCs) between the 4th and 8th passage. All cultures were incubated for two different time points (3 and 7 days). At each time point, the metabolic activity and viability of hDP-MSCs seeded (1 × 10^5^ cells/cm^2^) in 24-well plates TCP (with 150 mg of propolis), scaffolds (US and CU), and TCP plates (control) were evaluated using the MTT (3-(4,5-dimethylthiazol-2-yl)-2,5-diphenyltetrazolium bromide, Sigma) assay. Ten microliters of MTT solution (5 mg mL^−1^ in phosphate-buffered saline (PBS) was added and incubated for 3 h at 37 °C. Afterwards, the formed formazan salts were dissolved in dimethylsulfoxide (200 μL, Sigma, USA). Finally, the solution was transferred to a 96-well plate, and the absorbance was read at 550 nm (microplate spectrophotometer, MultiscanGo, Thermo Scientific, Waltham, MA, USA). The absorbance of the samples was normalized for the sample area and shown as a percentage respective to TCP wells.

#### 2.4.9. Statistical Analysis

All the experiments were performed in triplicate. Results were expressed as the mean ± standard deviation (SD). Statistical tests were performed using the software IBM SPSS Statistics V21 (IBM, Armonk, NY, USA). An unpaired Student’s *t*-test was used to evaluate the significance between experimental groups, and a value of *p* < 0.05 was considered statistically significant.

## 3. Results and Discussion

### 3.1. Wollastonite Particles, Filaments, and Scaffolds

**Figure 1:**
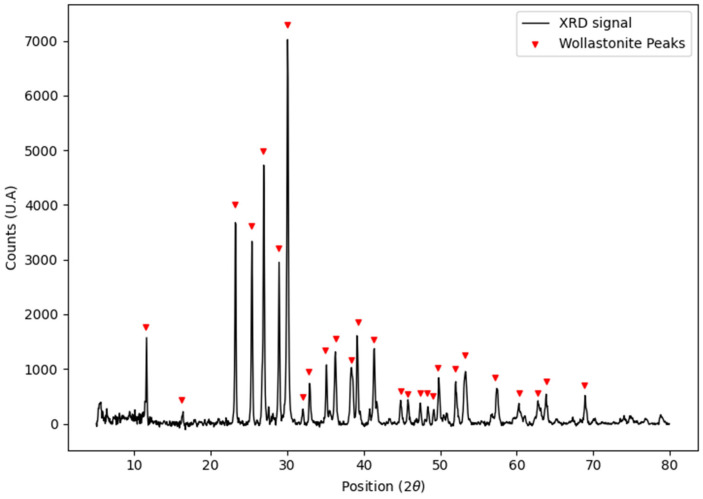
X-ray diffractogram of the wollastonite particles used to generate the compound for filament manufacturing.

Figure 1 shows the X-ray diffractogram of the wollastonite particles used to load the filaments.

The only crystalline phase identified in the diffractogram is wollastonite (Wollastonite-2\ITM\RG 00-027-0088). The filaments prepared with 20% *w*/*w* wollastonite in PLA are white, flexible, and have adequate size and texture for 3D printing.

**Figure 2:**
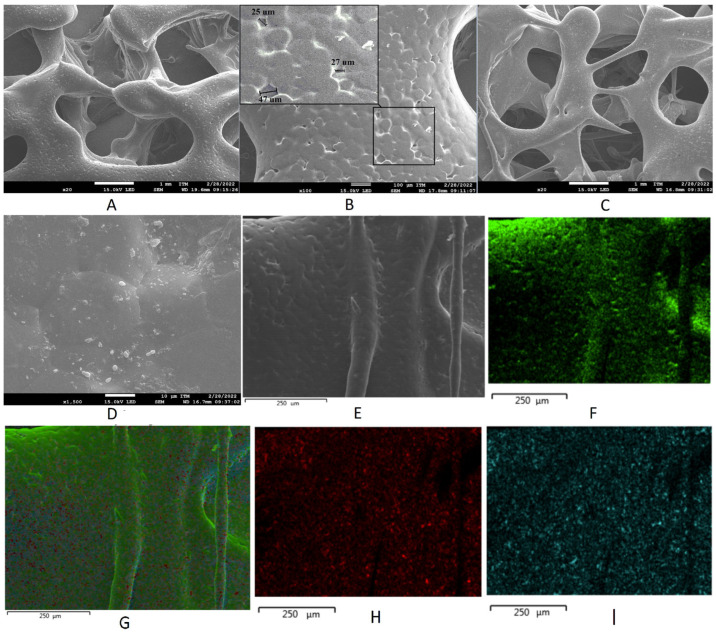
Scanning electron microscopy images of scaffolds (**A**,**B**) without particles and (**C**–**E**) with wollastonite particles. (**F**–**I**) Energy-dispersive X-ray spectroscopy elemental maps of scaffolds with wollastonite particles.

Figure 2 displays SEM photomicrographs of the scaffolds obtained for the PLA filament with and without 20% *w*/*w* wollastonite particles. Both scaffolds were successfully printed with a geometric shape of the gyroid TPMS used for their design. Herein, 1 cm scaffolds were printed with a pore volume of 64.5% and a pore diameter of approximately 1.4 ± 0.1 mm, which is adequate for cell anchorage and growth. Many works have shown that the growth of sizeable, organized cell communities requires the optimized porous structure of the scaffolds [45]. The minimum requirement for pore size is ~100 μm due to cell size, migration requirements, and transport. However, pore sizes > 300 μm are recommended due to enhanced new bone formation and the formation of capillaries [46,47,48,49]. The gyroid-TPMS geometric shape ensures the desired interconnected porosity for scaffolds. On the surface of the scaffolds printed with PLA without wollastonite particles, pores of approximately 28 ± 3 μm diameter appear. These pores are homogeneously distributed over the entire scaffold surface. The compositional silicon and calcium maps for the scaffolds containing wollastonite particles allow for the elucidation of the homogeneous distribution of the particles throughout the scaffold surface.

### 3.2. Scaffold Characterization: Hydrophobicity, Swelling, and Degradation

**Figure 3:**
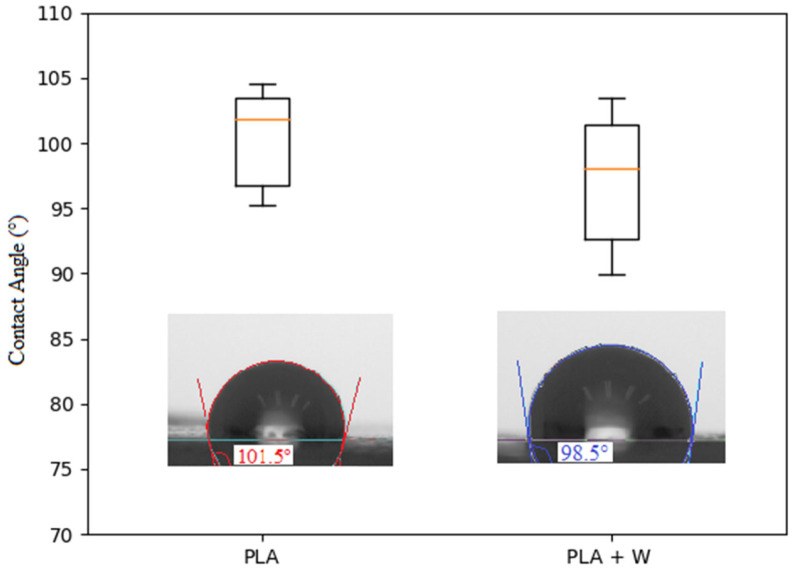
Contact-angle-test data for three-dimensional printed scaffolds with pure polylactic acid (PLA) and PLA with wollastonite particles.

Figure 3 presents the results from contact-angle measurements for PLA samples with and without particles. The pellets printed using PLA without particles display a slightly hydrophobic behavior. Although pellets with particles tend to have a slightly lower contact angle than those without particles, there are no substantial differences in the hydrophobicity between scaffolds with and without particles. PLA is a polymer that can be used to produce absorbable and biodegradable devices. PLA degrades as cells grow and organize and is absorbed by the body, leading to a natural replacement of the tissue [40]. The presence of particles in the compounds obtained does not affect the biological characteristics of the PLA. According to the technical data sheet provided by the manufacturer, the wollastonite particles mainly comprise acicular morphology particles with an average particle size (d_50_) of 3.5 μm and an asymmetric monomodal distribution. Unlike the materials obtained using the self-combustion method, materials synthesized by other methods do not show a high surface porosity, which could favor hydrophobicity [47,50,51]. 

**Figure 4:**
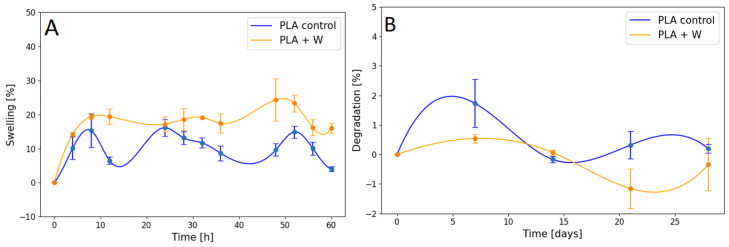
Graph of the (**A**) swelling ratio and (**B**) degradation properties of scaffolds prepared using PLA with and without wollastonite particles.

Figure 4 displays swelling and degradation graphs for scaffolds produced with and without wollastonite particles. After immersing the samples in the simulated body fluid (SBF) for 72 h, the scaffolds produced using PLA without wollastonite particles show slightly less swelling than those produced using PLA with wollastonite particles. Notably, the porosity rate reaches approximately 28 ± 3 μm on the walls of the scaffolds printed using PLA without wollastonite particles. The wollastonite particles partially occupy these pores, leaving some empty spaces among particle clusters and between particles and the polymeric material. The SBF lodges superficially both in the original pores and in the new pores formed owing to the presence of particles, generating a slightly higher swelling in scaffolds prepared using PLA with particles. However, scaffolds containing wollastonite particles suffer less degradation than those produced using PLA alone. Wollastonite particles are bioactive, indicating that in the presence of SBF, they form apatitic compounds that get deposited on their surfaces [52]. The mentioned compounds can be lodged in the original PLA pores, thereby decreasing the degradation calculated for the compounds.

### 3.3. Fourier-Transform Infrared Spectroscopy

**Figure 5:**
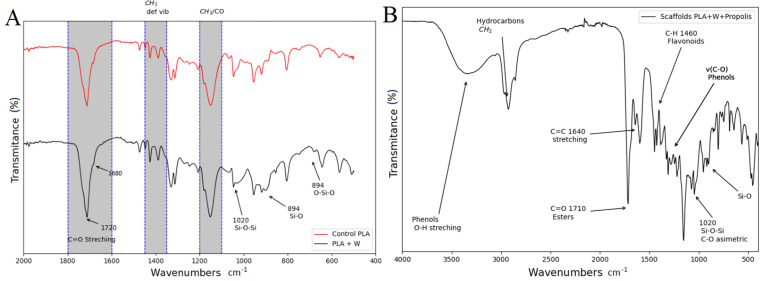
(**A**) Fourier-transform infrared (FTIR) spectrum for the PLA filament without particles (PLA control) and with wollastonite particles. (**B**) FTIR spectrum for the scaffold loaded with propolis using the impregnation method.

Figure 5A displays the FTIR spectra for both the PLA filament without particles (PLA control) and that with wollastonite particles. The spectrum for the PLA filament without particles exhibits a characteristic peak associated with the C=C bond at 1685 cm^−1^, a pronounced peak associated with the C=O bond at 1784 cm^−1^, vibrations associated with CH_3_ at 1389 and 1463 cm^−1^, and CH_3_/CO roll vibrations associated with the C–O–C stretches at 1113 and 1145 cm^−1^. The spectrum corresponding to the PLA filament with wollastonite particles contains vibration bands at 564, 894, and 1020 cm^−1^, which can be associated with the presence of wollastonite. The peaks at approximately 1020 cm^−1^ correspond to the Si–O–Si asymmetric stretching mode. The peaks at 894 cm^−1^ can be associated with the Si–O nonbridge bonds, and the absorption bands located at 682 and 564 cm^−1^ correspond to the Si–O–Si vibrational modes [53,54,55].

Figure 5B shows the FTIR spectrum corresponding to the scaffold loaded with propolis by impregnation. The broad, strong band at 3350 cm^−1^ occurs because of the phenolic group’s O–H stretching vibration or hydroxylated flavones. Spectral features related to phenols are also characterized by an interaction of O–H deformation and C–O stretching vibrations in the spectral range of 1405–1220 cm^−1^. The phenols are characterized by an aromatic ring C=C stretching at 1604 cm^−1^. The 1710-cm^−1^ peak corresponds to the carbonyl group (C=O) stretching vibrations of the ester bond and can be associated with saturated aliphatic esters in the propolis. C–H deformations and aromatic stretching at 1461 cm^−1^ are assigned to flavonoids and other medium- and weak-intensity absorption bands are attributed to the vibrations of several functional groups of phenols, flavonoids, and hydrocarbons [56,57,58].

### 3.4. Thermal Behavior

**Figure 6:**
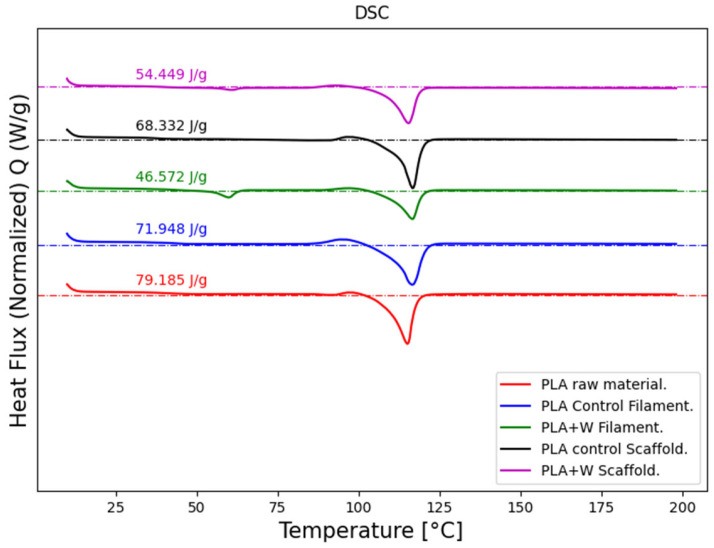
Differential scanning calorimetry profiles of the materials obtained at each processing stage conducted to produce PLA scaffolds with wollastonite particles.

To assess the effects of a processing stage on the characteristics of the material, DSC assessments were performed on the materials resulting from the stage. Figure 6 presents DSC graphs corresponding to the raw material (PLA), filament produced with PLA, PLA filament with wollastonite particles, and scaffolds printed using PLA with and without wollastonite particles. 

The crystallinity index and melting point of each material were calculated from the information captured from each DSC profile. The crystallinity index was determined using Equation (3), wherein Δ*H_exp_* represents the fusion enthalpy (J/g) determined using the DSC graph, Δ*H*_0_ is the theoretical enthalpy of fully crystalline PLA at 106 J/g [59,60], and *f* represents the PLA percentage by weight in each mixture [60].

(3) $$CI\;\left(\%\right)=\left(\frac{{\Delta H}_{exp}}{{\Delta H}_{0}\times f}\right)\times 100$$

Table 1 lists the melting points and Δ*H_exp_* and *CI* values for the materials obtained.

Here, the PLA melting point does not vary considerably regardless of the mechanical or thermal treatment conducted when producing the filaments, the scaffolds, or after adding wollastonite particles. This value is consistent with those reported in the literature [59,61].

The PLA crystallinity index decreases to a certain extent when subjecting the powdered raw material to temperatures of approximately 150 °C to form the particle-free filament. It decreases slightly more when printing the scaffold, which involves a temperature of approximately 160 °C. However, the differences in CI values between the filament and particle-free scaffold are insignificant, indicating that PLA is not thermally degraded in the manufacturing process. There is a slightly more significant reduction in the CI value of the processed filament with the wollastonite particles. It tends to rise when printing the material to produce the scaffold. Wollastonite particles are highly refractory, and their melting temperature is approximately 1500 °C [54], which causes some difficulties with the material filamentation process. However, printing the material is not difficult once the filament is obtained. The particles seem to contribute to improving the material’s thermal stability.

### 3.5. Mechanical Behavior

**Figure 7:**
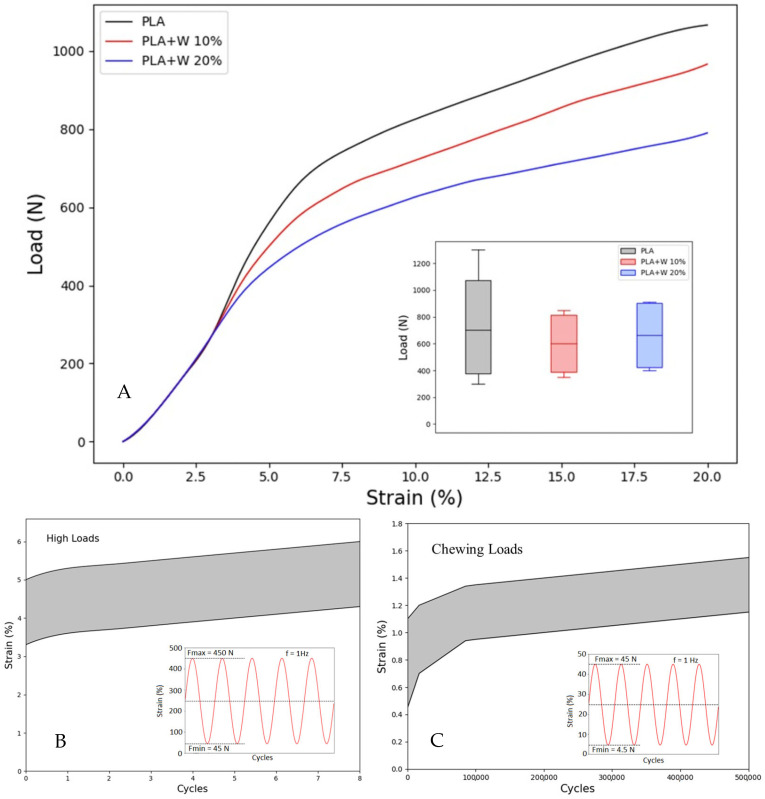
Mechanical behavior under static and cyclic loads. (**A**) Static response; the inset shows the box plot of the yield load for each condition, (**B**) cyclic under highs loads and (**C**) cyclic under masticatory loads; the insets show the cyclic loading conditions.

Figure 7 shows the average monotonic compressive response of the scaffolds with and without wollastonite particles. 

The initial elastic response of the samples shows a slope corresponding to Young’s modulus of the PLA matrix. The strain to reach yield in all the tested samples was close to 7.5%. Further, the inset to Figure 7A shows a box plot of the measured yield loads for various samples, where there are no statistically representative differences between the samples at an average value of F_y_ = 750 N.

A representative result of the cyclic tests at a high masticatory load of F_max_ = 450 N is shown in Figure 7B. Note that even for this load, which is well above the average masticatory load, the maximum strain reached after 8000 cycles is well below the yield strain (i.e., <7.5%), indicating that even under these stringent conditions, these scaffolds will be able to work correctly. Figure 7C further demonstrates representative cyclic loading results with a masticatory load similar to regular feeding masticatory loads (F_max_ = 45 N). Here, the maximum strain reached after 500,000 cycles is below 1.5% for the different samples tested, confirming the ability of the scaffolds to support high cycles of loading without failure.

The mechanical results confirm that at wollastonite particle fractions up to 20%, there is no significant reduction in the mechanical properties of the scaffolds compared to PLA scaffolds.

### 3.6. Biological Characterization

#### 3.6.1. Propolis Extracts

##### Total Phenols and Flavonoids

The characterization of propolis from different geographical areas has allowed the discovery of a wide variety of compounds and chemical profiles that include different types of phenols, flavonoids, aliphatic and aromatic acids, fatty acids, terpenes, saponins, tannins, lactones, alkaloids, and triterpenes, among others [62,63,64,65]. These compounds are usually associated with biological activities. Therefore, propolis extracts have been widely used in traditional medicine owing to their antioxidant, anti-inflammatory, analgesic, and antiproliferative properties [63,66,67,68]. 

The ethanolic extracts obtained in this study showed a total phenol content of 13.78 mg GAE g^−1^ EEP and a total flavonoid content of 5.03 mg QE g^−1^ EEP. These values are lower than those reported for Brazilian propolis (State of Paraná), wherein total phenol contents ranged from 48 to 76 mg GAE g^−1^ EEP and total flavonoid content ranged from 10 to 26 mg QE g^−1^ EEP [69]. However, propolis from Tame (Colombia) showed a behavior closer to that reported by Pobiega et al. in 2019 when assessing propolis from Poland (south central zone), wherein values from 11 to 16 mg QE g^−1^ EEP were obtained for total flavonoid content [70]. The quantification of organic compounds can be affected by the extraction methods and the organic solvents used to obtain the extracts [70,71]. The presence of phenols and flavonoids in propolis extracts may be related to antibacterial activity. Nevertheless, achieving a compositional chemical characterization may not guarantee the identification of the active ingredient because of the high compositional complexity and variability in propolis. The potential bactericidal effects reported for propolis may be associated with the presence of more than one component [72].

##### Antibacterial Activity of Propolis Extracts

The antibacterial activity was measured using three different methods: the determination of the MIC, measurement of the inhibition zones, and assessment of biofilm variability on the scaffolds. The extract obtained presented a MIC of 1.5 mg/mL against *S. aureus* and 2.0 mg/mL against *S. epidermidis*. The MIC obtained in this study proved to be much higher than that of the propolis evaluated in other regions of the world, such as those reported in [73], where propolis extracts obtained from Algeria (eastern zone) were studied, with MICs of 0.04 and 0.06 mg/mL against *S. aureus* and *S. epidermidis*, respectively [74,75,76,77]. Different types of antimicrobial synthetic and natural compounds have been described, as well as specific active mechanisms and MIC levels [74,75,76,77]. However, due to the complexity and variety of the compositional matrix found in propolis, there is no established level of MIC to use as an antimicrobial agent. Required propolis concentrations vary depending on the microbial species to be controlled and the developed products, but high concentrations might reduce the viability/proliferation of mammalian cells. These extracts were also used to assess EEPs against antibiotic-resistant strains, showing the neutralization of antibiotic resistance. Likewise, ethanolic extracts of red propolis from Brazil (Marechal Deodoro, Alagoas) have been assessed, obtaining a MIC of 0.256 mg/mL against *S. aureus*. These extracts showed a higher MIC than extracts from red propolis produced using chloroform and acetone [78]. The solvent used to obtain the propolis extracts directly affects their properties [78], broadening the options to acquire extracts with improved properties for specific applications. Future studies might consider changing solvents to reduce the MIC, with an antimicrobial potential over species related to osteomyelitis development. In this way, it will be possible to potentially control the growth of *S. aureus* and *S. epidermidis*, hence reducing the probability of implant failure associated with the development of chronic osteomyelitis by using EEP [79].

#### 3.6.2. Zone of Inhibition Assays with Propolis-Loaded Scaffolds

**Figure 8:**
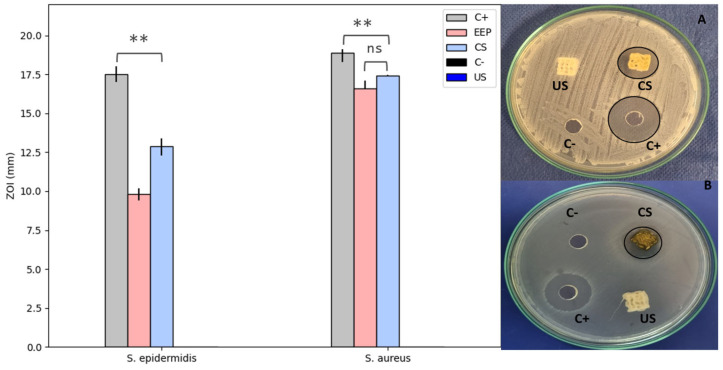
Zone of inhibition (in mm) of the PLA scaffolds impregnated with (CS) and without (US) ethanolic propolis extracts (EEP) against *S. aureus* (**A**) and *S. epidermidis* (**B**). Results are stat-ed as means ± SD (n = 3) (** *p* < 0.05, and (ns) points to non-significant difference).

The assay to evaluate the inhibition zones, conducted with scaffolds loaded with propolis extracts, showed a considerable EEP effect against the strains assessed. Compared to *S. epidermidis*, *S. aureus* exhibited greater sensitivity to propolis. Figure 8 shows the obtained inhibition zone (in mm). This result is consistent with the MIC values found against two bacterial strains. A higher MIC requires a higher concentration of propolis to achieve an evident effect on growth inhibition, as with *S. epidermidis*. Suggesting a relation between MIC and halo sizes, with a bigger inhibition zone in strains requiring a lower MIC. Besides the fact that the streptococci have phenol-soluble modulines (PSM) to counteract the antimicrobial effect of compounds similar to those present in EEP, these PSM vary between species, hence conferring different levels of antibiotic resistance [80].

The samples without propolis loads did not show inhibition halos, whereas those loaded with EEP exhibited halos of 17.42 ± 0.2 mm against *S. aureus* and 12.9 ± 0.5 mm against *S. epidermidis*. Herein, the propolis activity is significant but differs from the activity reported for chlorhexidine digluconate, which was used as a positive control. The results obtained in this study are consistent with those of several authors who have reported inhibition halos between 10 and 14 mm against *S. aureus* for propolis from Canada and Brazil [81,82,83].

In some cases, the antibacterial activity of propolis against *S. aureus* has been attributed to the presence of flavonoids, such as flavones, flavanols, flavonoids, and isoflavones [84]. The propolis assessed in this study comprised phenolic compounds and flavonoids, which could be related to the revealed antibacterial activity.

Propolis and some of its derivatives can affect the permeability of the cell membrane, thereby reducing the production of adenosine triphosphate, and thus, compromising bacterial motility and metabolism [85,86]. A reason for propolis generally being more effective against Gram-positive bacteria, such as those assessed in this study, is that these types of bacteria do not produce a high concentration of hydrolytic enzymes on their outer membrane (unlike Gram-negative bacteria), thereby avoiding counteracting the effects of propolis [87].

##### Viability of Bacterial Biofilms on Scaffolds

**Figure 9:**
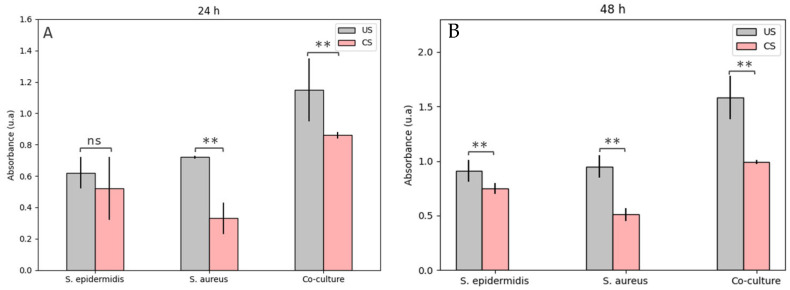
Viability of biofilms formed by *S. aureus*, *S. epidermidis*, and their coculture on the PLA and wollastonite scaffolds impregnated with (CS) and without (US) ethanolic propolis extracts af-ter (**A**) 24- and (**B**) 48-h culture. Results are stated as means ± SD (n = 3) (** *p* < 0.05, and (ns) points to non-significant difference).

Figure 9 illustrates the viability of biofilms formed by *S. aureus*, *S. epidermidis*, and their coculture on PLA and wollastonite scaffolds impregnated with (CS) and without (US) EEPs after 24 and 48 h culture. These results denote a decrease in the viability of microbiological growth in CS compared to US; the effect is noticeable after 24 and 48 h culture. In US, the growths of *S. aureus* and *S. epidermidis* are similar, although the coculture growth is substantially higher. This behavior denotes variations in the CS, where the growth of *S. aureus* is the most affected. However, in this group, significantly higher growth of the coculture is also observed. These results confirm that *S. aureus* is more susceptible to EEPs than *S. epidermidis*, a fact reported before [73].

*Staphylococci* species are characterized by genes encoding α-helix amphipathic peptides known as phenol-soluble modulins (PSMs). These PSMs are associated with pathogenic factors from some *Staphylococci*, such as *S. aureus* and *S. epidermidis*, because they are related to proinflammatory and cytolytic processes and can form biofilms in different tissues. However, they must be secreted in high concentrations to achieve the required colonization [88]. Given that the studied propolis contains phenolic compounds, they can be solubilized, allowing the bacterial strain to continue with its proliferation process and explicitly facilitating the action of the required adhesins in the first steps of biofilm formation [89]. It is worth mentioning that different types of PSMs are known, which may vary between different species of *Staphylococci*, thus allowing this genus to affect various mammalian tissues [80]. This explains that these species can increase their chances of forming biofilms and colonizing specific tissues due to their cocultures. In *S. epidermidis*, some PSMβ has been found to allow infectious factors to spread and form biofilms more quickly, making it less sensitive to specific antibacterial agents [80]. This also explains that greater IMC is required against the evaluated strains of *S. epidermidis*, and the inhibition halos were lower than those found against *S. aureus*. 

##### Cell Viability/Proliferation Assay

**Figure 10:**
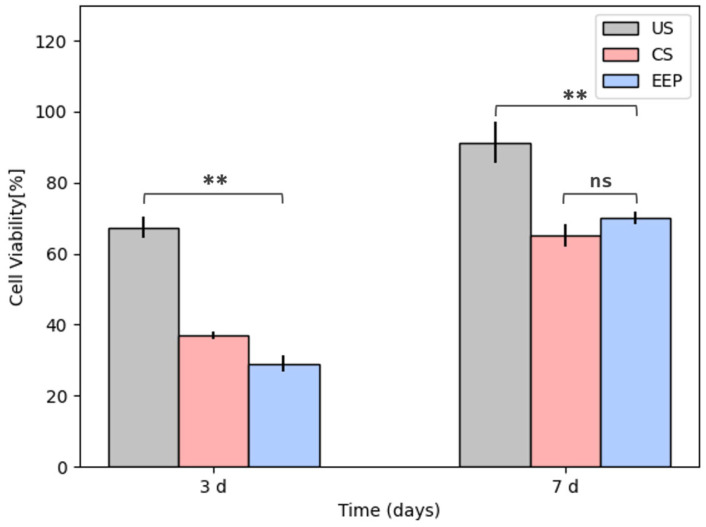
Cytotoxicity assay of a primary cell culture (Control) on the PLA and wollastonite scaffolds impregnated with (CS) and without (US) ethanolic propolis extracts (EEP) after 3 and 7 d culture. Results are stated as means ± SD (n = 3) (** *p* < 0.05, and (ns) points to non-significant difference).

Figure 10 shows the results from the viability/proliferation assay performed on the PLA and Wollastonite scaffolds with (CS) and without (US) propolis, along with those from EEP on a primary cell culture from a dental explant after 3 and 7 days of culture. The results showed a decreasing proliferation effect on the EEP wells and the propolis-loaded scaffolds at a 150 mg/mL concentration. The antimicrobial effect achieved with this EEP concentration was significant against the *S. aureus* and *S. epidermidis* cultures and their respective co-cultures. Further, the effect is more prominent after the first three days of culture. It might be related to the wax concentration on the EEP, which restricts the cellular proliferation on the surfaces containing them. However, after 7 days of culture, the inhibitory effect of the EEP on the primary cells culture becomes less severe, allowing the cellular viability to be above 50%. During the first 3 days of culture, the material by itself might have a detrimental effect on cell proliferation due to the observed 67% viability. This value turns out to be around double the value measured on the propolis samples (CS 37% and EEP 29%). After 7 days of culture, the material effect significantly reduces, allowing cell viability of 90%, while on the EEP samples, the antiproliferation effect gets reduced (CS 65% and EEP 70%). 

Studies on the viability/proliferation effects of propolis from different regions have shown that EEP has no cytotoxic effect after 24 h of culture on non-tumoral cells at concentrations up to 300 μg/mL. Nonetheless, these might present propolis sensibility after reaching 1000 μg/mL [90]. Propolis has also been used as an anti-proliferation agent in different kinds of tumoral cells, finding its effectiveness starting from doses of 50 μg/mL on breast cancer cell lines [91], prostate cancer cell lines [92], and doses of 250 μg/mL MCF-7 (human breast cancer), HT-29 (human colon adenocarcinoma), Caco-2 (colorectal epithelial adenocarcinoma) [91], and B16F1 (murine melanoma) [93].

The activity of propolis has been widely connected with the presence of phenols, flavonoids, and enzymes, which block specific steps in the proliferative process [91]. However, the propolis matrix is a complex compound, and some of its components might even affect non-tumoral cells. Furthermore, Salehi’s study argues that propolis might show some cytotoxic effect against non-tumoral cells such as L929, presenting a direct relationship between time and concentration for the viability/proliferation response. Still, this effect can be reduced by using encapsulation processes. Nevertheless, these processes require higher propolis concentrations to improve antimicrobial activity [94].

In this work, we did not encapsulate the propolis, so it was possible to evaluate the direct effect of EEP on the primary cell culture, finding that the effect is less aggressive after 7 days of culture. Future research might focus on reducing this undesired effect on cell proliferation during the first days of culture, keeping the antimicrobial effect against monospecific species and multi-species cultures as tested here.

## 4. Conclusions

PLA filaments produced using wollastonite particles were used to print scaffolds with a gyroid-TPMS geometric shape, which presented a pore volume of 64.5% and a pore diameter of 1.4 ± 0.1 mm. The wollastonite particles did not affect the scaffolds’ physical, mechanical, or thermal properties, which were successfully impregnated with ethanolic propolis extracts from Tame, Arauca (Colombia). These ethanolic propolis extracts exhibited a substantial antibacterial effect on the scaffolds against the monospecies cultures of *S. aureus* and *S. epidermidis* and their coculture, as well as an undesirable effect on viability/proliferation of hDP-MSCs in the short time that is reduced to 7 days of culture. The phenolic and flavonoid compounds found in the ethanolic propolis extracts cause the antibacterial effect. The propolis-impregnated scaffolds are an effective antibacterial agent in bone-implantation devices because they control species with a proliferative capacity for the biofilm-formation process required for severe infection processes.

## Figures and Tables

**Table 1:** Melting temperature, fusion enthalpy, and crystallinity index for different processing stages.

Material	Melting Temperature	Fusion Enthalphy (Δ*H_exp_*) J/g	Cristalinity Index (*CI*)
**PLA Raw Material**	115.03	79.185	74.703
**PLA Filament**	116.59	71.948	97.875
**PLA + W particles filament**	116.64	46.572	54.919
**PLA Scaffolds**	116.67	68.322	64.454
**PLA + W Particles Scaffolds**	115.39	54.449	64.209

## Data Availability

Not applicable.
